# Attachment and Entry of *Chlamydia* Have Distinct Requirements for Host Protein Disulfide Isomerase

**DOI:** 10.1371/journal.ppat.1000357

**Published:** 2009-04-03

**Authors:** Stephanie Abromaitis, Richard S. Stephens

**Affiliations:** Program in Infectious Diseases and Immunity, University of California Berkeley, Berkeley, California, United States of America; Duke University Medical Center, United States of America

## Abstract

*Chlamydia* is an obligate intracellular pathogen that causes a wide range of diseases in humans. Attachment and entry are key processes in infectivity and subsequent pathogenesis of *Chlamydia*, yet the mechanisms governing these interactions are unknown. It was recently shown that a cell line, CHO6, that is resistant to attachment, and thus infectivity, of multiple *Chlamydia* species has a defect in protein disulfide isomerase (PDI) N–terminal signal sequence processing. Ectopic expression of PDI in CHO6 cells led to restoration of *Chlamydia* attachment and infectivity; however, the mechanism leading to this recovery was not ascertained. To advance our understanding of the role of PDI in *Chlamydia* infection, we used RNA interference to establish that cellular PDI is essential for bacterial attachment to cells, making PDI the only host protein identified as necessary for attachment of multiple species of *Chlamydia*. Genetic complementation and PDI-specific inhibitors were used to determine that cell surface PDI enzymatic activity is required for bacterial entry into cells, but enzymatic function was not required for bacterial attachment. We further determined that it is a PDI-mediated reduction at the cell surface that triggers bacterial uptake. While PDI is necessary for *Chlamydia* attachment to cells, the bacteria do not appear to utilize plasma membrane–associated PDI as a receptor, suggesting that *Chlamydia* binds a cell surface protein that requires structural association with PDI. Our findings demonstrate that PDI has two essential and independent roles in the process of chlamydial infectivity: it is structurally required for chlamydial attachment, and the thiol-mediated oxido-reductive function of PDI is necessary for entry.

## Author Summary

*Chlamydia* is a large burden on global health. It is the most common cause of infectious blindness, and the CDC (Centers for Disease Control and Prevention) estimates that in the United States alone there are more than 2 million people with sexually transmitted *Chlamydia* infections. *Chlamydia* is an obligate intracellular bacteria; thus, attachment and subsequent invasion of cells are key steps in *Chlamydia* pathogenesis. While strides have been made in understanding the molecular mechanism of *Chlamydia* infection, fundamental aspects of this process still remain elusive. We have identified a host protein, protein disulfide isomerase (PDI), that is essential for *Chlamydia* attachment as well as for entry into cells. Cell-surface PDI-mediated disulfide reduction is required for *Chlamydia* entry into cells, whereas bacterial attachment is independent of PDI enzymatic activity. Although PDI is necessary for *Chlamydia* attachment, the bacteria apparently does not bind directly to cell-associated PDI, suggesting that *Chlamydia* attaches to a host protein(s) associated with PDI. This study advances our understanding of *Chlamydia* pathogenesis by the characterization of a host factor essential for independent stages of bacterial attachment and entry.

## Introduction

Fundamental to understanding of intracellular bacterial pathogenesis is knowledge of the mechanism of bacterial attachment and subsequent entry into cells. There are two main processes by which bacteria stimulate their entry into nonphagocytic cells: by bacterial contact mediated activation of a cell surface receptor (the “zipper” mechanism) or by injecting bacterial proteins into the cell cytosol (the “trigger” mechanism) [1],[2]. Once the bacterial and host factors involved in the invasion process are identified this knowledge can be employed to devise antimicrobial strategies that target cellular infection. Blockade of this first step of bacterial infection is ideal for intracellular bacteria as these pathogens are able to avoid a number of host defenses by residing within cells.

*Chlamydia* is an obligate intracellular bacteria that can infect a number of different eukaryotic cells. Human chlamydial infection causes a wide range of pathologies. *Chlamydia* is the most common bacterial sexually transmitted disease [3], the leading cause of infectious blindness [4], and a community acquired respiratory pathogen [5]. *Chlamydia* infects cells as a metabolically inactive elementary body (EB) and then once within cells differentiates into the metabolically active but noninfectious form known as the reticulate body (RB). The EB are small (0.3-µm) and have a rigid outer membrane consisting of a mesh of disulfide cross-linked cysteine-rich proteins [6]. This membrane structure causes the EB to be osmotically stable and thus resistant to the stresses of the extracellular environment [7]. The RB, which is much larger (1-µm), is not osmotically stable owing to a decrease in disulfide cross-linked envelope proteins. Following replication the RB condense back into EB in a process that involves the expression of EB-specific disulfide-rich proteins and oxido-reductive processing. These EB can then infect neighboring cells or new hosts.

Attachment and entry into cells are key steps in chlamydial development and pathogenesis, yet the mechanism governing these interactions is still unknown. A number of bacterial ligands, including the major outer membrane protein [8], glycosaminoglycans [9],[10], heat shock protein 70 [11], and OmcB [12],[13] have been implicated in the process. It is likely that a host proteinacious factor(s) is involved in *Chlamydia* attachment as infectivity is lost following mild trypsin treatment of cells [14]. Several host proteins including: epithelial membrane protein 2 [15], mannose 6-phosphate receptor [16], the estrogen receptor complex [17], platelet-derived growth factor receptor [18], and protein disulfide isomerase (PDI) [17],[19] influence *Chlamydia* attachment. However, only one mammalian protein, PDI, has been demonstrated to be involved in attachment of multiple species and serovars of *Chlamydia* [19].

The role for PDI in *Chlamydia* infection was originally elucidated by proteomic analysis of CHO6 cells. CHO6 cells were generated by chemical mutagenesis of *Chlamydia*-susceptible CHOK1 cells and the mutant cells are resistant to attachment of *C. trachomatis*, *C. pneumoniae*, and *C. psittaci* [20]. CHO6 have a defect in PDI processing and express two forms of the protein. CHO6 express full length PDI, which is found in the parental cell line CHOK1, as well as a truncated form lacking the N-terminal signal sequence that is unique to CHO6 cells [19]. This truncated form of PDI present in CHO6 is not the result of a mutation in PDI itself but is instead likely due to an unidentified processing defect in CHO6 cells [19]. It was determined that ectopic expression of full length PDI in CHO6 rescues chlamydial attachment and consequently infectivity [19].

PDI is a multi-functional protein: it catalyzes the reduction, oxidation, and isomerization of disulfide bonds, it can act as a chaperone or anti-chaperone [21], and PDI is a subunit of collagen prolyl 4-hydroxylase and microsomal triglyceride transferase [22]. PDI consists of five domains. It contains two thioredoxin-like catalytic domains (a and a′) separated by two non-catalytic domains (b and b′) and has a small C-terminal domain (c). The two catalytic domains contain characteristic CGHC active-site motifs, the cysteines in these sites are essential for PDI enzymatic activity [23]. An ER retention signal (KDEL) is located at the C-terminal of PDI, PDI also has an N-terminal signal sequence. PDI is highly enriched in the endoplasmic reticulum but is also found in the cytosol, nucleus, and on the cell surface [24]. PDI reductive function at the cell surface has been shown to be essential for entry of a number of different viruses [25]–[28], as well as being necessary for activating diphtheria toxin [29].

It is known that PDI is involved in chlamydial infection [19], but the precise role of PDI is not clear as PDI has a number of diverse functions in cells. In this study we evaluated the role of PDI enzymatic activity in *Chlamydia* infectivity. We have determined that although cellular PDI is required for both *Chlamydia* attachment and entry the requirement is mechanistically different in the two processes. PDI cell surface enzymatic activity was necessary for entry of bacteria into cells. In contrast *Chlamydia* attachment to host cells required PDI but was independent of cell surface PDI enzymatic activity.

## Results

### Cellular PDI is necessary for *Chlamydia* attachment

**Figure 1:**
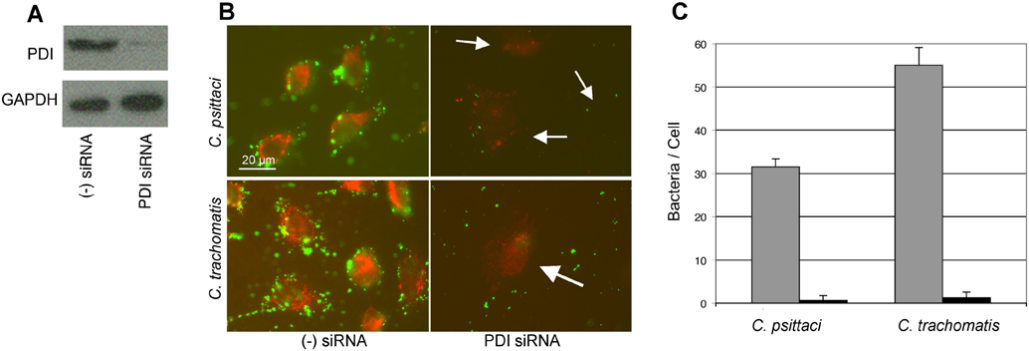
siRNA downregulation of PDI inhibits attachment of *Chlamydia*. (A) Immunoblot of cells transfected with PDI-targeted siRNA or non-targeting siRNA using antibody to PDI. GAPDH expression was evaluated to ensure equivalent loading. (B) Following siRNA treatment, *C. psittaci* and *C. trachomatis* attachment were examined. PDI staining is shown in red, and bacteria are stained green. Because of the significant PDI downregulation, cells in PDI siRNA panels are marked by arrows. siRNA downregulation of PDI led to a dramatic decrease in bacterial attachment, but attachment was not affected by treatment with non-targeting siRNA. (C) The number of bacteria attached to cells following treatment with non-targeting siRNA (gray) or PDI siRNA (black) was determined by quantification of the number of cell-associated bacteria by counting eight separate fields of view containing at least ten cells. Error bars indicate standard error of the mean (SEM).

CHO6 cells, which have a mutation that affects PDI processing [19], are resistant to attachment of multiple species of *Chlamydia* [20]. It has previously been shown that chlamydial infectivity of CHO6 can be rescued by expression of recombinant PDI [19], suggesting that PDI is involved in bacterial attachment. The mutation in CHO6 leading to differential processing of PDI and consequent lack of *Chlamydia* attachment is unknown. PDI is essential for cell viability, thus gene disruption approaches cannot be used to test if PDI is necessary for *Chlamydia* attachment or if additional mutations play a role in the phenotype of the CHO6 cell line. In contrast, siRNA has been used to transiently knockdown expression of PDI in mammalian cells [30]. siRNA-mediated downregulation of PDI was used to test whether PDI alone is required for *Chlamydia* infectivity. HeLa cells were transfected with PDI-targeting siRNA and PDI knockdown was assessed by immunoblot. Approximately 80–90% reduction in PDI expression was achieved (Figure 1A). When PDI was depleted from HeLa cells bacteria were no longer able to attach to and thus infect cells (Figure 1B). The degree of chlamydial attachment to siRNA treated cells was quantified by counting the number of bacteria attached to cells. Following PDI depletion there were only 0.69±1.04 *C. psittaci* and 1.27±1.39 *C. trachomatis* per cell, a 97.8% and 97.7% reduction in attachment respectively (Figure 1C). These results demonstrate that cellular PDI is essential for attachment of *Chlamydia* to the surface of host cells, making PDI the only host protein identified that is required for *Chlamydia* attachment.

### Enzymatically nonfunctional PDI can restore *Chlamydia* attachment but not entry into CHO6 cells

To further characterize the nature of the PDI requirement in *Chlamydia* infectivity, we tested the hypothesis that the oxido-reductive or cysteine isomerase roles of PDI were required for restoration of *Chlamydia* attachment and entry to CHO6 cells. The catalytic domain of PDI is well characterized and specific amino acids involved in PDI isomerase activity have been identified [31]. The role of PDI enzymatic activity in chlamydial infectivity was analyzed by generating recombinant PDI with disabled catalytic domains. This was accomplished by converting the four key cysteine residues within the catalytic domains to serine (PDI-4CS). Although PDI-4CS is no longer able to reduce, oxidize, or rearrange disulfide bonds it can still fulfill PDI chaperone, anti-chaperone, and structural roles [31].

**Figure 2:**
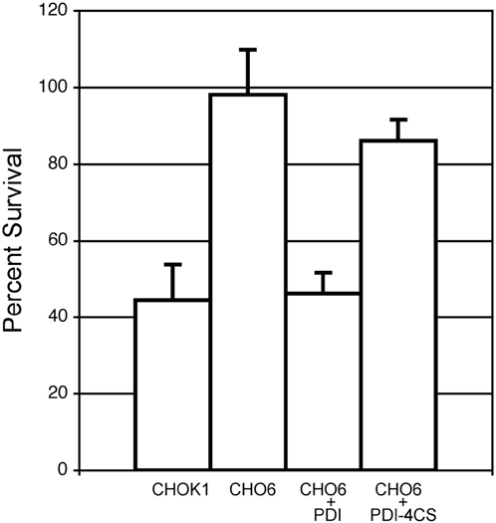
Cell surface PDI function is restored in CHO6+PDI but not in CHO6+PDI-4CS. Cell surface PDI enzymatic activity was assessed by diphtheria toxin (DT) sensitivity assays. Toxin killing of cells requires plasma membrane PDI function, percent survival was determined by comparing the level of cell viability in DT treated and untreated cells. Cells were incubated with 100 ng ml^−1^ DT, washed, and then incubated at 37°C. 72 h after toxin treatment, cell viability was determined by using the Promega CellTiter 96 Aqueous One Cell Proliferation Assay. CHOK1 cells were sensitive to DT, but CHO6 cells were not. When PDI was expressed in CHO6 (CHO6+PDI), the cells became sensitive to DT, indicating that cell surface PDI activity was restored in CHO6+PDI. Surface PDI activity is not restored in CHO6+PDI-4CS, as CHO6+PDI-4CS cells were relatively resistant to DT. Error bars indicate standard error of the mean (SEM).

Prior to examination of *Chlamydia* infectivity restoration in cells expressing PDI-4CS, cell surface PDI enzymatic activity was evaluated. Plasma membrane PDI activity can be tested by cellular sensitivity to diphtheria toxin (DT) as cell surface PDI enzymatic activity is required for DT-mediated killing of cells. PDI reduces interchain disulfide bonds in DT triggering chain separation that then allows for translocation of DT chain A across the membrane and subsequent toxin-induced cell death [29]. CHO6 cells have aberrant cell surface PDI activity and are resistant to DT. If native PDI is expressed in CHO6 cells toxin sensitivity is restored [19]. DT sensitivity of CHO6 expressing native PDI (CHO6+PDI) or CHO6 expressing enzymatically disabled PDI (CHO6+PDI-4CS) were tested. As previously reported, CHO6+PDI became sensitive to toxin (Figure 2), whereas CHO6 cells expressing PDI lacking enzymatic function (CHO6+PDI-4CS) were largely resistant to DT (Figure 2). These results demonstrated that cell surface PDI enzymatic activity is rescued in CHO6 following expression of native PDI but not in cells expressing PDI lacking enzymatic function. These data establish a model that can be used to specifically address the role of plasma membrane PDI enzymatic activity in *Chlamydia* infectivity.

**Figure 3:**
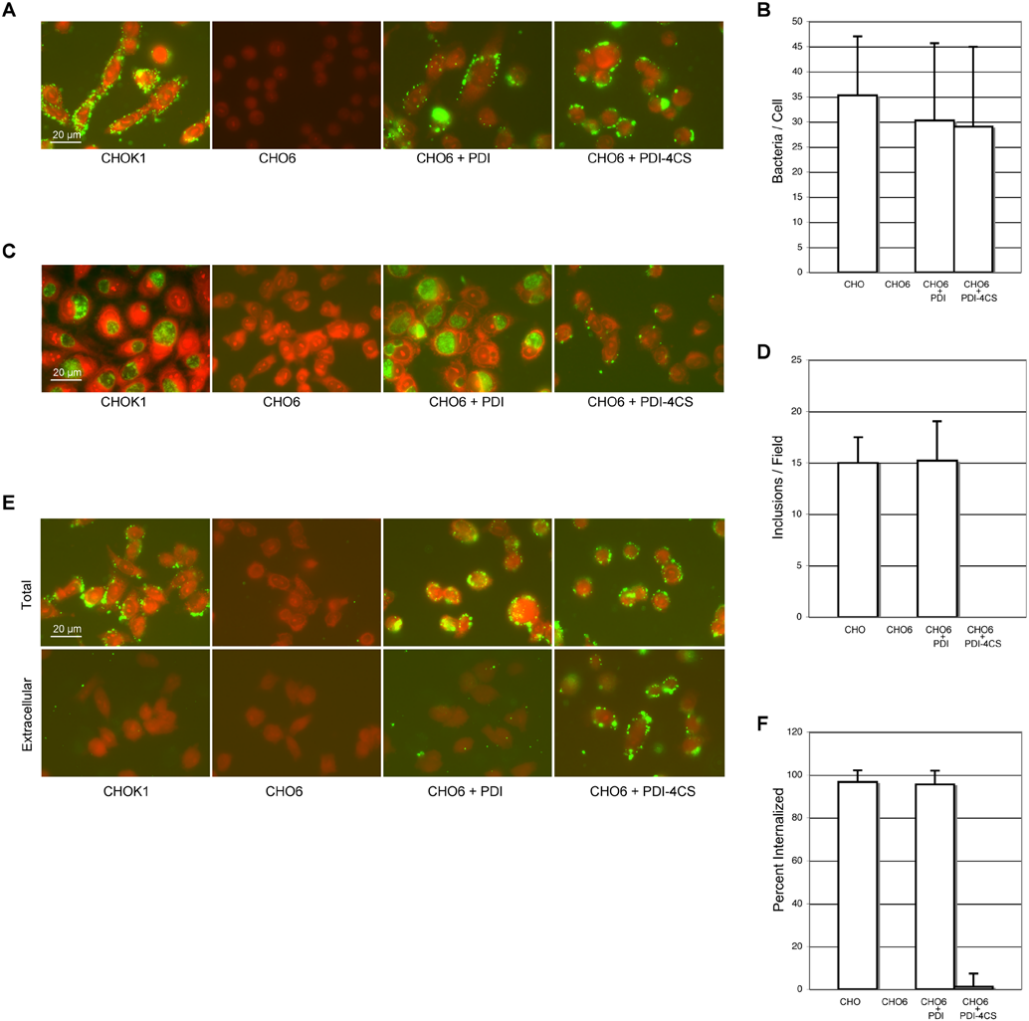
PDI enzymatic activity is necessary for bacterial entry but not for attachment. CHO6 cells were transfected with vectors expressing wildtype PDI (CHO6+PDI) or an enzymatic mutant of PDI lacking active site cysteine residues (CHO6+PDI-4CS). 48 h after transfection, cells were infected with *Chlamydia* for subsequent attachment and entry analysis. Cells were fixed and evaluated by immunofluorescence, bacteria are green and counter staining shown with Evans blue. (A) Bacterial attachment was analyzed 1 h post-infection. Bacterial attachment was recovered in CHO6+PDI as well as in CHO6+PDI-4CS, indicating that PDI enzymatic activity is not necessary for bacterial attachment. (B) The number of bacteria attached to cells was determined by quantification, the number of bacteria associated with each cell in eight separate fields of view containing at least ten cells. Error bars indicate standard of deviation (STDEV). (C) 24 h after infection *Chlamydia* infectivity was evaluated. Infectivity was restored in CHO6+PDI. No productive infection was observed in CHO6 cells or in CHO6+PDI-4CS. In CHO6+PDI-4CS, the bacteria remained persistently attached to the cell, indicative of the requirement for PDI enzymatic activity for bacterial entry. (D) Infection was quantified by counting the number of inclusions per field of view in eight separate fields of view containing at least ten cells. Error bars indicate the STDEV. (E) Cells were incubated at 37°C for 2 h to allow for bacterial entry. Entry was analyzed by comparing the total number of cell-associated bacteria for staining of permeabilized cells (Total) and the number of extracellular bacteria determined by staining unpermeabilized cells (Extracellular). Bacterial entry was recovered in CHO6 cells expressing PDI (CHO6+PDI) but not in CHO6 cells expressing enzymatically nonfunctional PDI (CHO6+PDI-4CS). (F) Percent internalization represents 1 minus the number of extracellular bacteria divided by the total number of bacteria multiplied by 100. The number of extracellular and total bacteria was determined by quantifying the number of bacteria associated with each cell per field of view in eight separate fields of view containing at least ten cells. Error bars indicate the STDEV.

CHO6 cells were transfected with vectors expressing native PDI or PDI-4CS and both attachment and entry were separately evaluated. Despite lacking PDI enzymatic activity at the cell surface, CHO6 cells transfected with PDI-4CS showed equivalent *Chlamydia* attachment as cells transfected with native PDI (Figure 3A). The level of attachment recovery was quantified by counting the number of bacteria associated with cells (Figure 3B). These data show that while PDI is necessary for chlamydial attachment, the function of PDI in attachment is independent of the protein's enzymatic activity.

Because PDI that is enzymatically nonfunctional could restore bacterial attachment to the cell, the ability of *Chlamydia* to establish a productive infection in cells expressing the parental or enzymatic mutant PDI protein was evaluated (Figure 3C). The development of *Chlamydia* laden vacuoles was only observed in the parental CHOK1 and CHO6 cells expressing PDI (Figure 3C). Infection rescue was quantified by determining the number of inclusion per field of view (Figure 3D), no inclusion were seen in CHO6 cells or CHO6 cells expressing PDI-4CS. It was noted that despite the lack of productive infection of CHO6+PDI-4CS, many of the bacteria remained persistently attached to the cells throughout the 24 h course of the experiment (Figure 3C).

Enzymatically nonfunctional PDI was capable of rescuing attachment but not infectivity. This outcome could be the result of an enzymatic role for PDI in either cellular entry or in other chlamydial developmental processes soon after entry that may prevent growth. Chlamydial entry was evaluated by allowing attached *Chlamydia* to enter cells by shifting them to 37° C for 2 h. Entry was analyzed by comparing the number of surface bound bacteria by immunofluorescence staining of unpermeabilized cells between CHO6+PDI versus CHO6+PDI-4CS. After 2 h at 37°C, entry was restored in CHO6+PDI with 95.7% of bacteria internalized following the 2 h incubation (Figure 3E and 3F). In contrast, there was no significant internalization of *Chlamydia* that had attached to CHO6+PDI-4CS (Figure 3E and 3F). Although PDI, independent of its enzymatic function was sufficient to restore chlamydial attachment, enzymatically functional PDI was required for uptake of bacteria into host cells.

From these results it can be concluded that PDI serves two distinct yet essential functions for *Chlamydia* attachment and entry. The role of PDI in attachment is not enzymatic but perhaps limited to a structural or chaperone function, whereas subsequent bacterial entry requires PDI enzymatic activity.

### Cell surface PDI enzymatic activity is necessary for *Chlamydia* entry into cells

Evaluation of *Chlamydia* infectivity in CHO6 cells expressing enzymatically nonfunctional PDI indicate that cell surface PDI activity is required for entry of bacteria but not for attachment. However, from those experiments it is equivocal if the PDI enzymatic activity necessary for bacterial entry is occurring at the cell surface or intracellularly. To specifically test if the required PDI enzymatic activity was occurring at the cell surface, *Chlamydia* attachment and entry were evaluated in CHOK1 cells in the presence of bacitracin. Bacitracin is a membrane impermeable PDI-specific inhibitor that has been used to test the role of plasma-membrane-specific function of PDI in mammalian cells [32]–[34]. Bacitracin is considered to be a PDI inhibitor because it does not inhibit thioredoxin mediated reduction [32]–[34]. The precise mechanism of bacitracin inhibition of PDI function is not known. Bacitracin has previously been used with *Chlamydia*. Davis *et al.* [17] found that addition of the inhibitor during infection led to a 16 to 36% decrease in bacterial infectivity. Given the dual role of PDI in *Chlamydia* infection illuminated by genetic approaches, we revisited these early findings by examining the effect of bacitracin-mediated inhibition of cell surface PDI on not only *Chlamydia* infection but also attachment.

**Figure 4:**
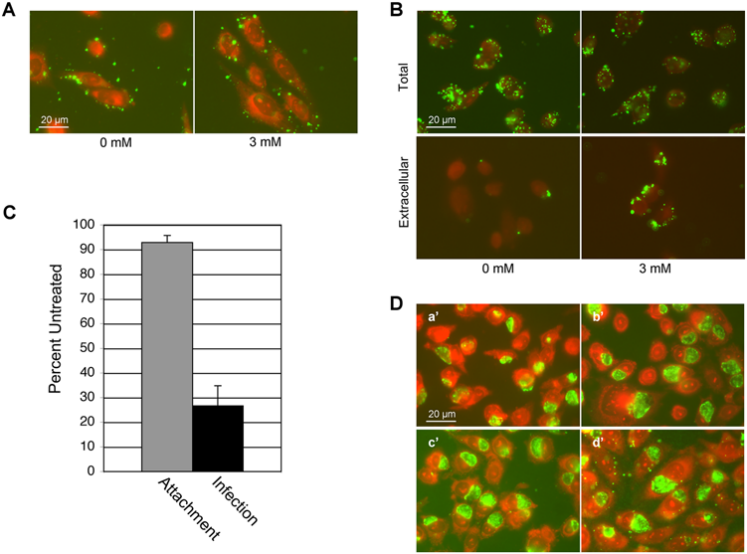
Confirmation that cell surface PDI enzymatic activity is necessary for bacterial entry using bacitracin. (A) *Chlamydia* attachment to CHOK1 was evaluated by immunofluorescence staining of bacteria (green) 1 h after infection and counter staining with Evans blue. Cells were infected and then maintained in media containing 0 mM or 3 mM bacitracin. Similar levels of bacterial attachment were observed with and without bacitracin. (B) Entry was examined after a 2 h incubation at 37°C. Entry was analyzed by comparing the total number of cell-associated bacteria (permeabilized) and the number of extracellular bacteria (unpermeabilized). 3 mM bacitracin inhibited bacterial entry. (C) The effect of bacitracin on *Chlamydia* attachment and infection was quantified and is shown as the % attachment and infection of cells not treated with bacitracin. Attachment was analyzed 1 h post-infection and infection was evaluated 24 h post-infection. (D) The effect of bacitracin on *Chlamydia* development was examined by evaluating cells 24 h post-infection that had not been treated with bacitracin (a′), been treated with 3 mM bacitracin for 20 min prior to infection (b′), been treated with bacitracin for the first 8 h of infection (c′), or been treated with bacitracin only for the last 16 h of the 24 h infection (d′). In all cases the development of bacteria-containing vacuoles was seen, indicative of a normal infection.

CHOK1 cells were inoculated in the presence of bacitracin and attachment was then evaluated. In the presence of bacitracin *Chlamydia* attached to cells at similar levels as untreated cells (Figure 4A). This demonstrates that extracellular PDI enzymatic function is not required for chlamydial attachment and supports the results of our experiments with CHO6 expressing enzymatically nonfunctional PDI (Figure 3A and 3B). The role of plasma membrane PDI function in *Chlamydia* entry was examined by inoculating cells and cultivating them in media containing bacitracin for 2 h. When bacitracin was present during initial infection and for the 2 h following attachment *Chlamydia* were unable to enter cells and EB remained persistently attached to cells (Figure 4B). This confirmed that there is a requirement for plasma membrane PDI activity for chlamydial entry and supports our previous analyses. The effect of bacitracin on bacterial attachment and infection was quantified using a quantitative real-time PCR assay as described previously [35]. When bacitracin was present the level of attachment was 92.9% that of untreated cells, indicating that cell surface PDI enzymatic activity is not necessary for *Chlamydia* attachment (Figure 4C). In contrast when the level of infection was quantified 24 h after bacterial attachment, infection of bacitracin treated cells was reduced by 73.4% as compared to untreated cells (Figure 4C).

Along with being a PDI-specific inhibitor, bacitracin is also a bactericidal antibiotic that targets gram-positive bacteria. The mechanism of bacitracin-mediated bacterial death is that it inhibits bacterial cell wall synthesis by inhibiting dephosphorylation of lipid pyrophosphate. *Chlamydia* is a gram negative-like bacteria, but it remained important to ensure that the block of bacterial entry by bacitracin was not simply due to damage to chlamydial organisms by the inhibitor treatment. We determined that treating cells with the inhibitor only prior to infection and followed by washing had no effect on *Chlamydia* attachment or entry (Figure 4D). Normal bacterial development was also seen if the inhibitor was removed or added after the first 8 h of the infection (Figure 4D). The reversibility of the inhibition demonstrates that the observed effects were not due to damage to *Chlamydia* or the cell by the inhibitor treatment. To ensure that the requirement for PDI enzymatic activity was not unique to CHOK1 cells attachment and infectivity analysis with bacitracin was also conducted in HeLa cells. As in CHOK1 cells *Chlamydia* attached to HeLa cells in the presence of bacitracin but were unable to enter (Figure S1).

Using genetics we determined that PDI lacking enzymatic function was able to complement attachment but not bacterial entry and subsequent development. DT analysis demonstrated that following complementation with enzymatically nonfunctional PDI there was a defect in cell surface PDI activity. The ability of bacitracin to inhibit bacterial entry, in addition to our results following PDI expression in CHO6, define an essential role at the cell surface for PDI enzymatic function in *Chlamydia* entry.

### Cell surface PDI-mediated reduction triggers *Chlamydia* entry into cells

**Figure 5:**
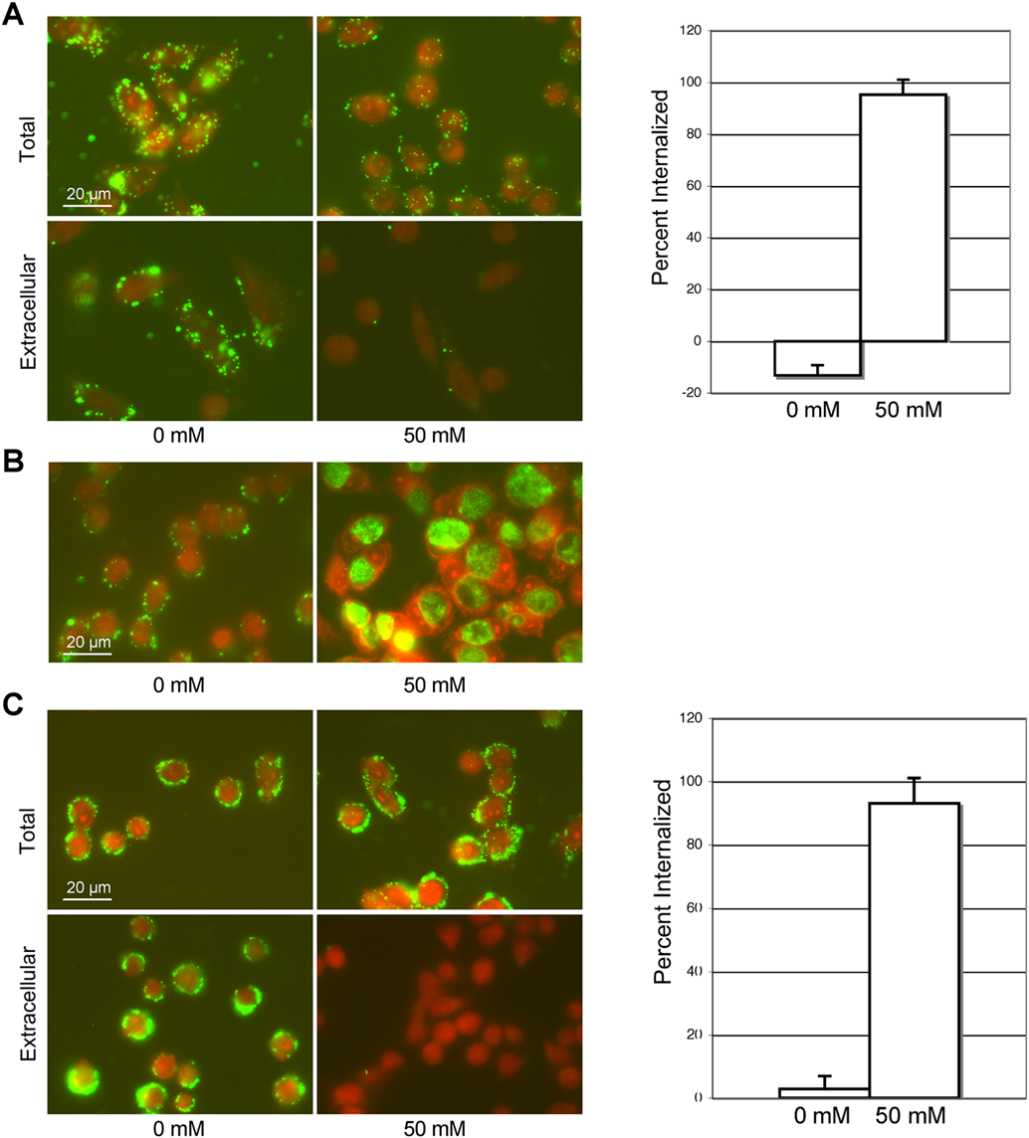
Cell surface PDI reductive function is necessary for bacterial entry. (A) Bacterial entry into CHOK1 cells treated with 3 mM bacitracin was analyzed after a 2 h incubation at 37°C. Bacteria are shown in green and counter stained with Evans blue. Entry was assessed by comparing the total number of cell-associated bacteria (permeabilized) and the number of extracellular bacteria (unpermeabilized) in cells that were treated with 0 mM or 50 mM TCEP. Percent internalization represents 1 minus the number of extracellular bacteria divided by the total number of bacteria multiplied by 100. The number of extracellular and total bacteria was determined by quantifying the number of bacteria associated with each cell per field of view in eight separate fields of view containing at least ten cells. Error bars indicate the STDEV. Bacitracin-mediated inhibition was overcome by the addition of the membrane-impermeable reducing agent TCEP. (B) Infection was evaluated in CHOK1 cells treated with 3 mM bacitracin 24 h post-infection in cells that had been treated for the initial 2 h of infection with 0 mM or 50 mM TCEP. Development of a normal infection occurred in cells treated with TCEP, whereas the bacteria remained persistently attached to the outside of untreated cells. (C) Bacterial entry into CHO6 cells transfected with a vector expressing enzymatically nonfunctional PDI (PDI-4CS) was analyzed after a 2 h incubation at 37°C. Percent internalization was determined as in (A). Bacterial entry is seen in cells treated with 50 mM TCEP but not in untreated cells.

Having established that cell surface PDI enzymatic activity was required for *Chlamydia* entry into cells we next sought to determine the molecular mechanism of that activity. PDI is able to reduce, oxidize, and rearrange disulfide bonds and all three of these activities are arrested in the presence of bacitracin. Because of the role of PDI-mediated reduction in viral entry [25]–[28], we evaluated if it was a reductive function that was necessary for *Chlamydia* entry into cells. CHOK1 cells were infected with *Chlamydia* in the presence of bacitracin, arresting infectivity of the bacteria at the cell surface. The membrane impermeant disulfide reducing agent TCEP (Tris(2-carboxyethyl)phosphine hydrochloride) was then added and the cells were incubated at 37° C for 2 h. The addition of TCEP was able to overcome bacitracin inhibition of *Chlamydia* entry, confirming that the stimulus necessary for bacterial entry into cells is a PDI-mediated reduction occurring at the cell surface (Figure 5A). Following entry and TCEP removal *Chlamydia* were able to establish a productive infection in cells demonstrating that the bacteria were not damaged by the 2 h incubation with TCEP (Figure 5B).

The mechanism of bacitracin mediated inhibition of PDI enzymatic activity is not known, making it possible that TCEP was simply inhibiting the interaction between PDI and bacitracin and in that manner restoring bacterial entry. To control for this possibility the effect of TCEP on entry of *Chlamydia* into CHO6 cells expressing enzymatically nonfunctional PDI (CHO6+PDI-4CS) was analyzed. *Chlamydia* are able to attach to CHO6 cells expressing PDI-4CS but the bacteria are unable to enter (Figure 3). When TCEP was added the bacteria were then able to enter the CHO6 cells expressing non-enzymatically functional PDI (Figure 5C). From these experiments we can conclude that it is cell surface PDI mediated reduction that is required for *Chlamydia* uptake into cells.

### *Chlamydia* does not directly attach to cell surface PDI

**Figure 6:**
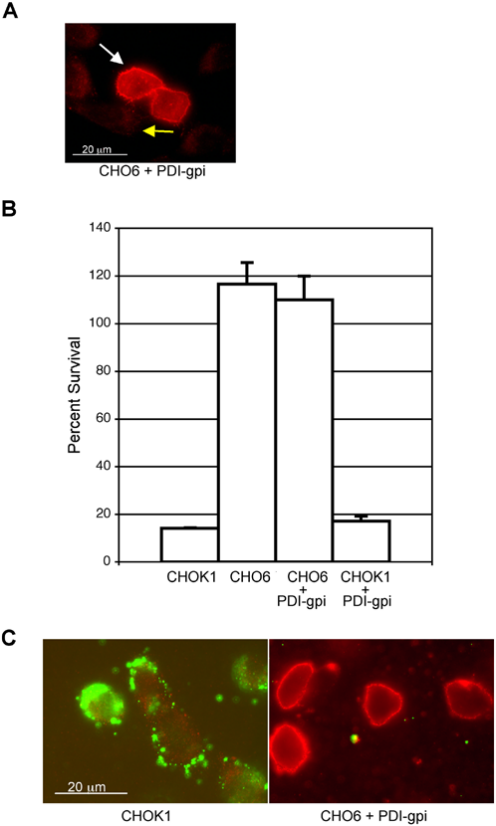
PDI interaction with other cell surface proteins is necessary to rescue bacterial attachment to CHO6 cells. (A) CHO6 cells transfected with a vector expressing PDI with a gpi-anchor (PDI-gpi) are marked with a white arrow, untransfected cells are indicated by a yellow arrow. Cells were fixed and stained for PDI using PDI-specific antibody. Expression of PDI-gpi led to a large amount of PDI localized at the cell surface. (B) Cell survival was analyzed 72 h after DT treatment. CHOK1 cells were extremely sensitive to toxin, whereas CHO6 cells were relatively resistant. Expression of PDI-gpi in CHOK1 or CHO6 cells did not alter toxin sensitivity, indicative of an inability of PDI-gpi to properly interact with other cell surface proteins. (C) *Chlamydia* attachment to CHOK1 and CHO6+PDI-gpi was analyzed, bacteria are shown in green, and PDI is stained red. There was no bacterial attachment to CHO6+PDI-gpi.

The functional role of PDI in the bacterial attachment stage was next examined. The most direct hypothesis is that *Chlamydia* bind PDI as a receptor. PDI is not an integral membrane protein. PDI is secreted from cells and then maintained at the surface through electrostatic interactions with other cell surface proteins [24]. To test for *Chlamydia* binding directly to cell surface PDI we generated a PDI protein that was tethered to the plasma membrane by a C-terminal gpi anchor. A similar strategy has been used to study the role of PDI in HIV entry [28]. This plasma membrane anchored PDI (PDI-gpi) was expressed in CHO6 cells and resulted in high-level cell surface PDI expression that was readily detectable by PDI-specific antibody (Figure 6A).

Prior to analysis of bacterial attachment to CHO6 expressing PDI-gpi, it was first determined if the gpi-anchored PDI was able to interact with other cell surface proteins in the same manner as unanchored PDI. This was tested by evaluating DT sensitivity of cell expressing PDI-gpi. PDI interaction with DT bound to its cell surface receptor (heparin-binding epidermal growth factor) is necessary for DT mediated cell death [36]. Whereas toxin sensitivity is recovered by expression of unanchored PDI (Figure 2), the gpi-anchored PDI was unable to normally interact with other cell surface proteins and could not restore DT sensitivity to CHO6 cells (Figure 6B). The high-level of PDI-gpi expression did not interfere with normal cell surface interaction, as it did not reduce CHOK1 toxin sensitivity (Figure 6B). From these results we could conclude that CHO6 expressing PDI-gpi functioned as a model for testing if *Chlamydia* was able to directly attach to PDI independent of PDI interactions with other cell surface proteins. *Chlamydia* attachment to CHO6 cells expressing PDI-gpi was evaluated, no recovery of bacterial attachment was observed (Figure 6C). These results suggest there is a lack of *Chlamydia* binding directly to PDI.

**Figure 7:**
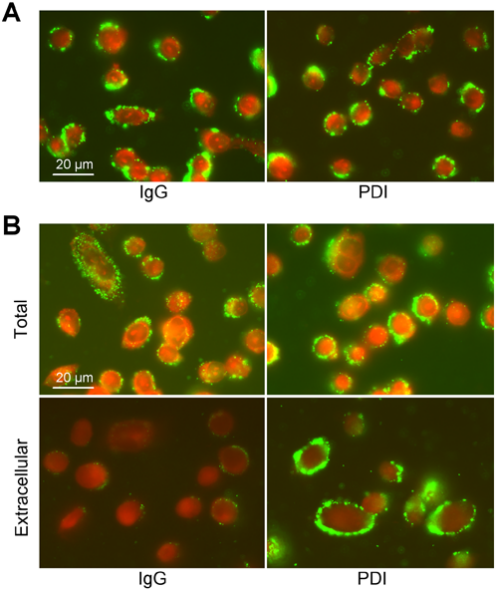
PDI-specific antibody inhibits *Chlamydia* entry but not bacterial attachment. (A) Cells were incubated with 100 µg ml^−1^ polyclonal PDI-specific antibody or 100 µg ml^−1^ irrelevant IgG antibody prior to and during bacterial attachment. Bacteria were allowed to attach for 1 h and binding was analyzed, bacteria are shown in green and counter stain is Evans blue. There was no significant difference in the amount of bacteria that bound to cell with and without PDI antibody treatment. (B) Entry was analyzed after a 2 h incubation at 37°C. Entry was assessed by comparing the total number of cell-associated bacteria (permeabilized) and the number of extracellular bacteria (unpermeabilized) in cells that were treated with100 µg ml^−1^ polyclonal PDI-specific antibody or 100 µg ml^−1^ irrelevant IgG antibody.

The experiments with the gpi-anchored PDI are indicative of a lack of direct attachment on *Chlamydia* to PDI, but the possibility that the gpi-anchor was causing a structural change in PDI that led to the lack of bacterial attachment could not be ruled out. To address this possibility the effect of polyclonal PDI-specific antibody of *Chlamydia* attachment was analyzed. The effect of antibody to PDI on *Chlamydia* infection has previously been evaluated by Davis *et al.* [17], they observed a temperature dependent decrease in *Chlamydia* infection. We further developed those findings by specifically addressing inhibition of bacterial attachment as well as entry. When PDI antibody was present prior to and during bacterial attachment no significant change in the number of bacteria attached to cells was observed (Figure 7A), suggesting that *Chlamydia* was not directly binding PDI. Bacterial entry in the presence of PDI antibody was also evaluated. The antibody significantly reduced bacterial entry (Figure 7B) and led to a persistent attachment phenotype similar to what was seen in the presence of bacitracin (Figure 4). The inhibition of *Chlamydia* entry by PDI antibody corroborated our previous determination that cell surface PDI reductive function was required for bacterial uptake.

A polyclonal PDI antibody was used in these experiments, and it is likely that this antibody would inhibit PDI enzymatic activity by steric interference. To directly assess the level of PDI enzymatic activity a turbidimetric assay of insulin disulfide reduction was performed (Protocol S1). We determined that addition of PDI antibody to the reaction significantly reduced the rate and level of insulin reduction, indicating that the antibody had an inhibitory effect of PDI enzymatic activity (Figure S2). We conclude that while cellular PDI is necessary for *Chlamydia* attachment to cells the bacteria does not initiate attachment through a direct interaction with PDI. Much like HIV and diphtheria toxin, *Chlamydia* likely binds to a cell surface protein(s) that is associated with PDI.

## Discussion

Cell surface PDI-mediated disulfide bond reduction is involved in the infectious entry of a number of viruses. Upon binding of human immunodeficiency virus (HIV) envelope protein to CD4 receptor and co-receptor (CCR5 or CXCR4) PDI reduces disulfide bonds in HIV gp120, exposing the gp41 fusion peptide [28]. The fusion peptide then inserts into the target cell surface triggering viral and cell membrane fusion. For Newcastle disease virus, cell surface PDI enzymatic activity is required for the conformational changes in the viral fusion protein that are necessary for cell-viral membrane fusion [27]. Similarly PDI-mediated reduction of Sindbis virus envelope is required for membrane fusion and release of the viral genome into cells [25]. We have experimentally established that the initial stages of chlamydial infectivity, attachment and entry, each require host cell surface PDI; however, the functional participation of PDI was mechanistically unique at each stage. Cell surface PDI enzymatic activity was required for *Chlamydia* entry. Independent of PDI enzymatic function, PDI was additionally essential for bacterial attachment to host cells.

The role for protein disulfide exchange in chlamydial infection has been previously explored using inhibitors. Davis *et al.* [17] and Raulston *et al.* [11] showed that inhibition of cell surface reductive function by addition of bacitracin or dithio-bis-2-nitrobenzoic acid (DTNB) adversely affected infectivity of *C. trachomatis* serovar E. These results can now be understood in terms of the direct enzymatic role for PDI in bacterial entry. Raulston *et al.* [11] found no inhibition of bacterial attachment using DTNB similar to our observations with bacitracin. When Davis *et al.* [17] and Raulston *et al.* [11] evaluated the effect of bacitracin or DTNB they reported a slight decrease in infectivity, whereas we observed a near complete loss of bacterial entry and subsequent infection. These differences are likely due to the fact that PDI is constitutively trafficked to the cell surface [24], making bacitracin inhibition functionally reversible. Both Davis *et al.* [17] and Raulston *et al.* [11] removed the inhibitor after initial bacterial attachment allowing newly exported PDI to stimulate uptake. In our experimental design, the inhibitor was present throughout the course of the infection and if the inhibitor was removed following attachment, our results were similar to those of Davis *et al.* [17] and Raulston *et al.* [11]. Our data also significantly expand the previous observations as this now extends the requirement for disulfide exchange in the process of bacterial infection from one strain to multiple species of *Chlamydia*.

A requirement for PDI in chlamydial attachment was implicated by complementation of the PDI gene in attachment-deficient mutant CHO6 cells [19]. We anticipated that the oxido-reductive enzymatic activity of PDI would be required for bacterial attachment, similar to what is characterized for viral attachment [25],[27],[28]. It was a surprise that chlamydial attachment to CHO6 cells was rescued by complementation with the enzymatically inactive PDI-4CS. Further evaluation using PDI inhibitors confirmed that *Chlamydia* attachment is independent of cell surface PDI enzymatic activity. Testing of gpi-anchored PDI tethered to the cell surface suggested that chlamydiae do not directly bind PDI as the sole target for attachment and this implicates an interaction with other proteins that require PDI. Consistent with characterized functions of PDI, it may be PDI's function as a chaperone or structural component of a host protein or protein complex [37] that is required for chlamydial attachment. PDI chaperone activity is independent of the protein's two catalytic domains (a, a′) and PDI functions as a chaperone outside of the ER [38],[39]. PDI could serve to stabilize a host receptor protein in the correct orientation or context for bacterial binding. There are also examples of PDI functioning as a structural subunit of proteins. PDI is the β-subunit of tetrameric enzyme collagen prolyl 4-hydroxylase, and PDI also makes up half of the heterodimeric protein complex microsomal triglyceride transfer protein [40],[41]. The receptor that *Chlamydia* binds may be a multiprotein complex that includes PDI.

In addition to a requirement of PDI for chlamydial attachment, it was shown that PDI enzymatic activity was necessary and sufficient to stimulate chlamydial entry into the host cell. Given the ability of simple chemical reduction to replace the enzymatic function of PDI for cell-adherent chlamydiae, it appears that reduction rather than disulfide exchange is minimally required for chlamydial entry. It is not known whether PDI functions to reduce a host or bacterial component to initiate entry. It has been previously shown that reduction of *C. trachomatis* L2 EB prior to infection leads to a decrease in inclusion forming units [42]. This experiment illustrates the problem of differentiating the effect of reduction on the infectious process. One can pre-reduce host cells or bacteria prior to infection, but due to the rapid rate of reoxidation following the reducing agent removal step and unknown detrimental effects, especially following pan-blocking of disulfides by chemical agents, make interpretation of outcomes confounded and uncertain.

One can speculate that following initial bacterial attachment, PDI enzymatic activity mediates the establishment of a functional contact between the chlamydial organism and the host cell leading to bacterial uptake. It is known that plasma membrane PDI interacts with a number of host surface proteins. For example, PDI modifies the adhesion receptors integrin α_2_β_1_ and L-selectin resulting in receptor activation and ligand binding [33]. As well as modifying cellular surface proteins, PDI binds and modifies various viral proteins and toxins [25]–[29]. Thus, there is biological precedent supporting PDI modification of either host or microbial factors required for pathogenesis.

Multiple cell surface proteins have been previously implicated in *Chlamydia* infectivity, these include epithelial membrane protein 2 [15], mannose 6-phosphate receptor [16], estrogen receptor complex [17], and platelet-derived growth factor receptor [18]. Unlike PDI, these proteins appear to be involved in the infectivity of only a subset of chlamydial species or biovars. PDI interacts with a broad array of host proteins within the endoplasmic reticulum and at the cell surface allowing for the possibility that PDI may be an underlying requirement for several independent *Chlamydia*-host cell receptor interactions. PDI is involved in protein folding in the endoplasmic reticulum and PDI expression can be correlated to the level of secretion of a number of proteins [43]–[45]. We established using PDI-specific chemical inhibitors, anti-PDI antibodies, and chemical reduction of bound organisms that the function of PDI in chamydial infectivity requires surface accessible PDI. Proteomic analysis using 2-dimensional gel electrophoresis of biotin-labeled surface proteins failed to show any detectible and consistent difference between CHO6 and CHOK1 cell lines other than for PDI (19 and data not shown). This suggests that the lack of bacterial attachment to CHO6 cells is not due to a general defect in plasma membrane protein composition. If the enzymatic role of PDI is targeted to a host protein it seems likely that this occurs at the cell surface and not in a secretion pathway.

Alternative to acting on a host protein, PDI could be targeting the chlamydial organism. The highly disulfide cross-linked structure of the chlamydial EB surface proteins that are only present in the infectious form of the chlamydial organism [7], suggest that these could be the target for PDI. It has been shown that reduction of the EB surface is necessary for surface display of bacterial Hsp70 [11] and it is possible that PDI activates changes in chlamydial surface proteins that are required to initiate cellular entry. The chlamydial protein Tarp is translocated into host cells following bacterial attachment [46]. This translocation occurs via type III secretion and triggers actin recruitment to the site of bacterial attachment [46]. Tarp is localized within EB and is not surface exposed until the commencement of bacterial entry [46]. PDI-mediated reduction of EB surface proteins may be essential to activate *Chlamydia* type III secretion into the cell membrane and subsequent Tarp translocation. The type III secretion system needle protein of *C. trachomatis*, CdsF, was recently identified [47]. CdsF is conserved among the chlamydial species, and it is one of the few bacterial needle proteins that contains cysteine residues. One can speculate that PDI-mediated reduction of the EB surface is involved in CdsF function.

While the full intricacies of the *Chlamydia*-host cell interaction remain enigmatic these findings illuminate important new details of the molecular mechanisms involved. *Chlamydia* attachment and entry into cells are separable processes that both have a unique requirement for PDI. Determination of the target of PDI enzymatic activity that leads to chlamydial entry may be expected to provide targets for generation of new anti-*Chlamydia* therapies and illuminate fundamental cell biological processes exploited by *Chlamydia* to mediate pathogen attachment and infectivity.

## Materials and Methods

### Cell lines, *Chlamydia*

CHOK1 and CHO6 cells were maintained in RPMI 1640 (Invitrogen, Carlsbad, CA) supplemented with 10% heat-inactivated fetal bovine serum (FBS) (Hyclone, Logan, UT), 2 mM glutamine (Invitrogen), and 1 mM HEPES (Invitrogen). HeLa and L929 cells were maintained in RPMI 1640 supplemented with 10% FBS. Cells were grown at 37°C in an atmosphere containing 5% CO_2_. *C. trachomatis* L2/434/Bu EB and *C. psittaci* PF6 BC were purified from L929 cells on 30% and 30–44% discontinuous Renografin gradients (E. R. Squibb and Sons, Cranbury, NJ) [48] and stored at −80°C until use.

### *Chlamydia* attachment and infectivity

On the night prior to analysis 1×10^5^ cells were plated on 12 mm glass coverslips in 24 well plates. Cells were washed with Hanks buffered saline solution (HBSS) (Invitrogen) and then incubated with *C. trachomatis* L2/434/Bu or *C. psittaci* PF6 BC in RPMI 1640 with 10% FBS for 1 h at 24°C [19], [49]–[52]. Following infection cells were washed 4 times with HBSS and methanol fixed for attachment analysis. For entry and infectivity analysis fresh cell culture media was added and cells were incubated 2 h for entry and 24 h for infectivity at 37°C in an atmosphere containing 5% CO_2_. For entry analysis cells were fixed with methanol or with 4% paraformaldehyde, for infectivity assessment cells were fixed with methanol. Following fixation coverslips were incubated 15 min in blocking solution (HBSS+2.5% bovine serum albumin (BSA)) (Fisher, Fair Lawn, NJ). Blocking solution was removed and coverslips were incubated 1 h with mouse anti-*Chlamydia* MOMP antibody for *C. trachomatis* or anti-*Chlamydia* LPS antibody (Santa Cruz Biotechnology, Santa Cruz, CA) for *C. psittaci* and then washed with HBSS. The wash was followed with a 30 min incubation with goat anti-mouse AlexaFluor 488 (Invitrogen) and Evans Blue. Coverslips were mounted in VectaShield Hard Set mounting media (Fisher) and evaluated on a Nikon Eclipse E800 microscope. When used bacitracin (Sigma, St. Louis, MO) was added 20 min prior to infection at 3 mM, infection was performed in media containing 3 mM bacitracin and following infection cells were maintained in culture media with 3 mM bacitracin. When used 50 mM TCEP (Pierce, Rockford, IL) in cell culture media was added following bacterial attachment. For attachment and entry inhibition analysis cells were incubated for 20 min with polyclonal rabbit anti-PDI antibody (Stressgen Victoria, BC) or goat anti-bovine IgG antibody (Pierce, Rockford, IL), the bacterial inoculum was then added to the antibody containing media and cell were incubated for 1 h at 24°C. When Stressgen rabbit anti-PDI antibody was used for PDI visualization, PDI staining was performed similarly to *Chlamydia* staining and goat anti-rabbit AlexaFluor 594 (Invitrogen) was used as a secondary.

For quantification the number of cell-associated apple-green fluorescing particles of size and shape consistent with 300 nM organisms were counted in two planes in 8 separate fields of view containing at least 10 cells. Fluorescent particles that appeared to be *Chlamydia* but were larger due to aggregation were enumerated separately when separate organisms could be discerned or when distinction was not possible were counted as one to provide a conservative estimate of bound organisms per cell.

### Protein disulfide isomerase expression and silencing

The gene encoding protein disulfide isomerase was cloned from CHOK1 cDNA and inserted into the expression vector pBICEP-CMV-1 (Sigma) yielding PDI with a N-terminal FLAG tag. PDI protein lacking enzymatic function (PDI-4CS) was generated by mutating 4 essential cysteine residues to serine with the Stratagene (La Jolla, CA) QuikChange Kit and primers Cys-55,58-Ser F (5′ - TGC CCC GTG GTC TGG CCA CTC CAA AGC TCT GG - 3′), Cys-55,58-Ser R (5′ - CCA GAG CTT TGG AGT GGC CAG ACC ACG GGG CA - 3′), Cys-399,402-Ser F (5′ - TAT GCC CCC TGG TCT GGC CAC TCC AAG CAG CT - 3′), and Cys-399,402-Ser R (5′ - AGC TGC TTG GAG TGG CCA GAC CAG GGG GCA TA - 3′). The gpi-anchor for PDI was amplified from human folate receptor 1 using primers GPI F (5′ - ATT GCC CGG GGC TGC AGC CAT GAG TGG - 3′) and GPI R (5′ – CAA TGT CGA CTC AGC TGA GCA GCC ACA GCA – 3′). The gpi-anchor was than ligated to PDI lacking a stop codon and the PDI-gpi construct was ligated into the pBICEP-CMV-1 vector. PDI vectors were transfected into CHO cells with Effectene (Qiagen, Valencia, CA) following manufacturer's instructions. *Chlamydia* attachment and entry were evaluated 48 h after transfection.

PDI was silenced in HeLa cells with PDI-specific siRNA (5′- GAC CTC CCC TTC AAA GTT GTT –3′) (Dharmacon, Layafette, CO) [30]. Dharmacon siControl non-targeting siRNA #1 was used at as a negative control. Cells were transfected as described in Hybiske and Stephens, 2007 with slight modification [35]. Cells were first transfected in 6 well plates with 10 µl of 40 µM siRNA duplexes with 5 µl of Olgiofectamine (Invitrogen) in OptiMem (Invitrogen). 24 h after transfection cells were transfected again. 90 h after the first transfection cells were replated on fibronectin (Sigma) coated coverslips in 24 well plates, and attachment, infectivity, or protein expression was evaluated 6 h after replating.

### Diphtheria toxin sensitivity

24 h prior to toxin treatment cells were plated in 96-well plates at 1×10^4^ cells per well. Cells were washed once with HBSS and than incubated for 4 h at 37°C with diphtheria toxin (Biomol, Plymouth Meeting, PA) (0 or 100 ng ml^−1^) in HBSS containing 15% FBS. Following incubation cells were washed three times with HBSS and were than maintain in RPMI 1640 without phenol red supplemented with 10% FBS, 2 mM glutamine, and 1 mM Hepes at 37°C in an atmosphere containing 5% CO_2_. 72 h after toxin treatment cell viability was determined by Promega (Madison, WI) CellTiter 96 Aqueous One Cell Proliferation Assay as directed by the manufacturer's instructions. Cell viability is linearly proportional to absorbance.

### Quantitative *Chlamydia* infectivity assay

The number of *Chlamydia* per cell was determined using a quantitative PCR based method previously described by Hybiske and Stephens, [35]. Genomic DNA from CHOK1 cells infected with *C. trachomatis* was isolated using the High-Pure PCR template preparation kit (Roche, Indianapolis, IN). Purified genomic DNA was used as a template in quantitative PCR to determine the relative levels of chlamydial (16S) and CHOK1 (ß-globin) genomic equivalents.

### Accession numbers

The NCBI Entrez (http://www.ncbi.nlm.nih.gov/sites/entrez?db=protein) accession number for genes discussed in this paper are *Homo sapiens* PDI (NP_006840) and *Cricetulus griseus* PDI (AAM_00284).

## Supporting Information

Figure S1 Bacitracin does not affect *Chlamydia* attachment to HeLa cells, but it completely inhibits subsequent bacterial infection. Prior to infection, HeLa cells were incubated 20 min with 0 mM or 3 mM bacitracin. Cells were then infected with *Chlamydia* in media with or without bacitracin. Addition of bacitracin had no significant effect on bacterial attachment (top panel). Bacterial infection was analyzed 24 h after initial inoculation. In cells cultured without bacitracin, the development of a productive infection, as indicated by the presence of large bacteria containing vacuoles, was observed (bottom panel). When bacitracin was present throughout the course of the infection, the bacteria remained persistently attached to cells and no productive infection occurred (bottom panel). These results are similar to our analysis of the effect of bacitracin on *Chlamydia* infection of CHOK1 cells (Figure 4).
(2.88 MB TIF)

Figure S2 Polyclonal PDI antibody inhibits PDI enzymatic activity. PDI enzymatic activity was evaluated by measuring the rate of insulin reduction spectrophotometrically at 650 nm as turbidity formation from the precipitation of the insulin B chain following insulin reduction. A control reaction (Control) was performed without PDI. A reaction with PDI (PDI) and with PDI preincubated with PDI-specific antibody (PDI+Ab) was also done. Readings were taken every 5 min for 1 h. Error bars indicate the standard of deviation from three separate experiments performed on the same day.
(0.57 MB TIF)

Protocol S1 Supporting Protocol
(0.03 MB DOC)
