# Supplementary figures and images for: Attachment and Entry of Chlamydia Have Distinct Requirements for Host Protein Disulfide Isomerase
Source: PLoS Pathog. 2009 Apr 3;5(4):e1000357. doi: 10.1371/journal.ppat.1000357 (PMC2655716; doi:10.1371/journal.ppat.1000357)

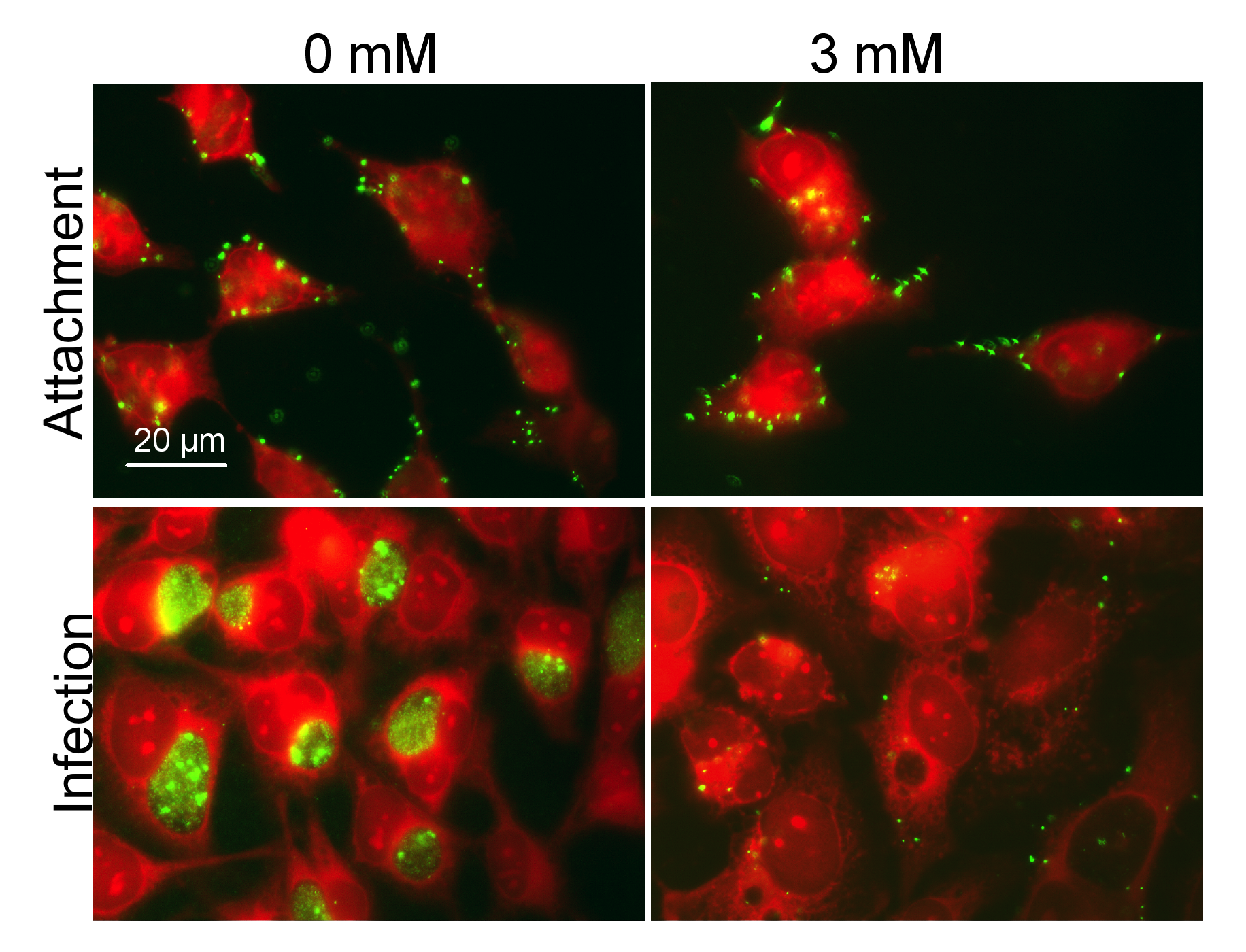

Supplement: Figure S1 — Bacitracin does not affect Chlamydia attachment to HeLa cells, but it completely inhibits subsequent bacterial infection. Prior to infection, HeLa cells were incubated 20 min with 0 mM or 3 mM bacitracin. Cells were then infected with Chlamydia in media with or without bacitracin. Addition of bacitracin had no significant effect on bacterial attachment (top panel). Bacterial infection was analyzed 24 h after initial inoculation. In cells cultured without bacitracin, the development of a productive infection, as indicated by the presence of large bacteria containing vacuoles, was observed (bottom panel). When bacitracin was present throughout the course of the infection, the bacteria remained persistently attached to cells and no productive infection occurred (bottom panel). These results are similar to our analysis of the effect of bacitracin on Chlamydia infection of CHOK1 cells (Figure 4). (2.88 MB TIF) [file ppat.1000357.s001.tif]

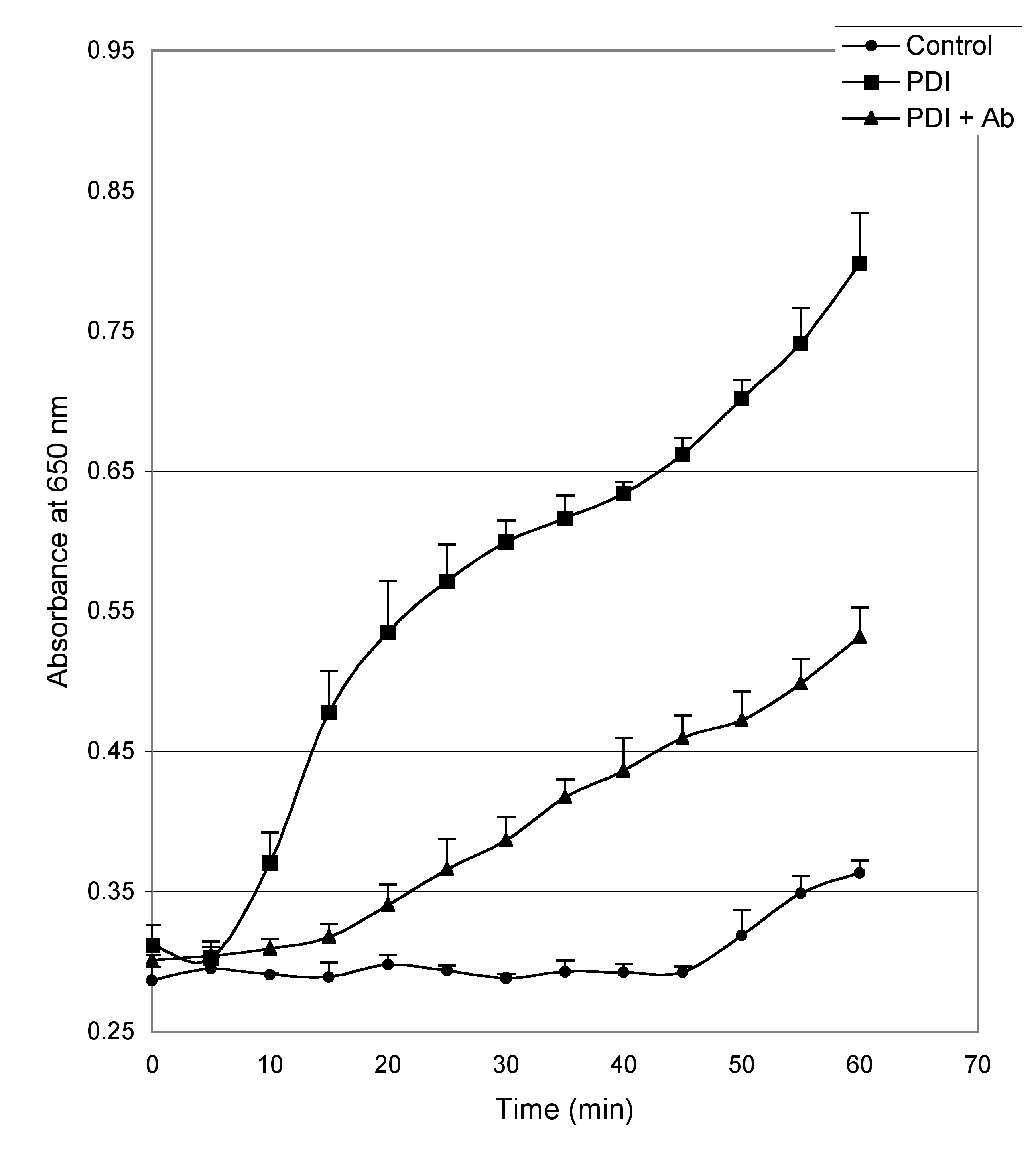

Supplement: Figure S2 — Polyclonal PDI antibody inhibits PDI enzymatic activity. PDI enzymatic activity was evaluated by measuring the rate of insulin reduction spectrophotometrically at 650 nm as turbidity formation from the precipitation of the insulin B chain following insulin reduction. A control reaction (Control) was performed without PDI. A reaction with PDI (PDI) and with PDI preincubated with PDI-specific antibody (PDI+Ab) was also done. Readings were taken every 5 min for 1 h. Error bars indicate the standard of deviation from three separate experiments performed on the same day. (0.57 MB TIF) [file ppat.1000357.s002.tif]
