# Supplementary material for: Attachment and Entry of Chlamydia Have Distinct Requirements for Host Protein Disulfide Isomerase
Source: PLoS Pathog. 2009 Apr 3;5(4):e1000357. doi: 10.1371/journal.ppat.1000357 (PMC2655716; doi:10.1371/journal.ppat.1000357)
Supplement: Protocol S1 — Supporting Protocol (0.03 MB DOC) [file ppat.1000357.s003.doc]

**Supporting Text:**

#### To directly assess the level of PDI enzymatic activity a turbidimetric assay of insulin disulfide reduction was performed. It is known that PDI catalyzes insulin disulfide reduction, and measurement of precipitation of the free insulin B chain had been used to directly asses PDI enzymatic activity [1, 2]. We observed high level PDI enzymatic activity within 10 min of reaction initiation by DTT addition (Figure S2). When PDI was excluded from the reaction no significant insulin reduction was observed with in the first 55 min of the experiment (Figure S2). Addition of PDI antibody to the reaction significantly reduced the rate and level of insulin reduction, indicating that the antibody had an inhibitory effect on PDI enzymatic activity (Figure S2).

**Supporting Protocol:**

__20μm__

#### Turbidimetric PDI activity assay

All reagents were prepared fresh on the day of the experiment. Insulin solution was prepared as described by Holgren (1979). Reactions contained 0.1 M potassium chloride pH 7.0, 2 mM EDTA, and 0.167 mM insulin from bovine pancreas (Sigma) in a total of 700 μl. When used PDI from bovine liver (Sigma) and polyclonal PDI antibody (Stressgen) were added at 10 μg and 40 μg respectively. The reaction was initiated by addition of 2 mM DTT. Measurements of absorbance at 650 nm were performed every 5 min for 1 h using a Pharmacia Biotech Ultrospec 3000.

#### Supporting References

1. Holmgren A: **Thioredoxin catalyzes the reduction of insulin disulfides by dithiothreitol and dihydrolipoamide**. *J Biol Chem* 1979, **254**(19):9627-9632.

2. Lundstrom J, Holmgren A: **Protein disulfide-isomerase is a substrate for thioredoxin reductase and has thioredoxin-like activity**. *J Biol Chem* 1990, **265**(16):9114-9120.
